# Temporal Factors Modulate Haloperidol-Induced Conditioned Catalepsy

**DOI:** 10.3389/fnbeh.2021.713512

**Published:** 2021-07-02

**Authors:** Lucía Cárcel, Luis G. De la Casa

**Affiliations:** Laboratory of Animal Behavior and Neuroscience, Department of Experimental Psychology, University of Seville, Seville, Spain

**Keywords:** classical conditioning, haloperidol, catalepsy, simultaneous conditioning, inter stimulus interval

## Abstract

Repeated pairings of a neutral context and the effects of haloperidol give rise to conditioned catalepsy when the context is subsequently presented in a drug-free test. In order to confirm whether this response is based on Pavlovian processes, we conducted two experiments involving two manipulations that affect conditioning intensity in classical conditioning procedures: time of joint exposure to the conditioned and the unconditioned stimulus, and the length of the inter-stimulus interval (ISI). The results revealed that both an increase in the length of context-drug pairings during conditioning and a reduced ISI between drug administration and context exposure increased conditioned catalepsy. These results are discussed in terms of the temporal peculiarities of those procedures that involve drugs as the unconditioned stimulus along with the role of Pavlovian conditioning in context-dependent catalepsy.

## Introduction

The oldest antecedents of the study of classical conditioning using drugs as Unconditioned Stimuli (US) can be traced back to experiments by I.P. Pavlov (1849–1936), who reported that repeated administration of morphine in the presence of the same context resulted in Conditioned Responses (CR) similar to those produced by the drug itself (Pavlov, 1927). Based on these pioneering studies, numerous investigations have been conducted demonstrating the conditioning of various responses produced by a wide range of drugs, and revealing that sometimes the CR was similar to that induced by the drug (e.g., Battisti et al., 2000; Mena and De la Casa, 2013), while in other cases the CR was opposite to the unconditioned effect of the drug (e.g., Siegel, 1975; Solomon, 1980). To explain these differences, Eikelboom and Stewart (1982) proposed that on some occasions the Conditioned Stimulus (CS) would be associated with a response that depends on the central nervous system, while others times it would be associated with a peripheral response which appears to compensate the effect of the drug. In the first case, the association between the drug and the CS results in a CR that is similar to that produced by the drug. However, in the second case, the CR is opposite to the response induced by the drug at the central level, and there is ample evidence showing that conditioning of such opponent response is governed by Pavlovian processes (e.g., Tiffany and Baker, 1981; Paletta and Wagner, 1986).

A particularly interesting case in the field of Pavlovian conditioning using drugs as USs is conditioning produced by pairing a neutral stimulus (typically a new experimental context) with the effects of haloperidol. This antipsychotic drug produces extrapyramidal side effects such as parkinsonism, akinesia, and acute dystonia (Lanis and Schmidt, 2001; Oliveira et al., 2016) that are related to a decrease in dopamine transmission in the striatal areas caused by the blockade of D2 receptors (Klemm, 1989; Dias et al., 2012; Barroca et al., 2019). The complexity of the conditioning process using haloperidol as the US is shown in experiments in which opposing results appear depending on the dose of the drug and type of test used. More specifically, in some experiments using the so-called “bar test,” a CR of catalepsy is observed with the animals maintaining unusual postures for long periods in a drug-free trial conducted after conditioning (e.g., Amtage and Schmidt, 2003; Banasikowski and Beninger, 2012; Oliveira et al., 2016). On other occasion in which spontaneous movement over an extended period is recorded in a drug-free test after conditioning, an increase in conditioned spontaneous locomotor activity has been observed (Dias et al., 2012; De la Casa et al., 2018, 2020). These contradictory findings can be accommodated by a proposal put forward by Dias et al. (2012), who point out that, depending on the administered dose, haloperidol can lead to both an increase and decrease in locomotor activity. Thus, when a low dose of haloperidol (0.03 mg/kg) is repeatedly administered, a progressive increase in the locomotor response is observed; however, when the administered dose is higher, a reduction in locomotor behavior induces the state of catalepsy (e.g., Nowak et al., 1988; Banasikowski and Beninger, 2012; Oliveira et al., 2016). Accordingly, in those experiments analyzing conditioning with haloperidol as the US, different results have emerged depending on the dose of drug administered during conditioning. Thus, with doses of haloperidol that we can classify as high (specifically 0.1, 0.25, and 0.5 mg/kg), a conditioned catalepsy effect has been observed on the drug-free test trial (Banasikowski and Beninger, 2012). In contrast, Dias et al. (2012) found an increase in conditioned locomotor activity after ten CS-context pairings and a low dose of haloperidol (0.03 mg/kg).

Irrespective of the registered CR (catalepsy or increased activity), it plays a relevant role in preparing the animal for the appearance of the US, and an issue of particular relevance in this area is to identify those processes that determine responses observed after repeated pairings of the drug and the context. Previous results in this field of research indicate that classical conditioning is responsible for the responses observed on the drug-free test. Thus, for instance, Amtage and Schmidt (2003) programmed eight conditioning trials consisting of daily injections of haloperidol (0.25 mg/kg) followed by catalepsy tests 60 min later. As expected, an increased catalepsy response emerged throughout the trials due to a sensitization effect. On Day 9, the animals were divided into two groups, one that received five extinction days in which the catalepsy test was preceded by a saline injection, and another group that received no experimental treatment. Finally, on a drug-free test day, catalepsy did not appear in the extinction group, whereas the effect was evident for the group that had not received exposure to the context-CS without the drug.

The purpose of the following experiments was to obtain additional evidence of the classical conditioning processes that underlie haloperidol-induced catalepsy in a drug-free situation. To this end, we tested whether certain parameters that affect Pavlovian conditioning with more conventional USs have the same effect on conditioning using haloperidol as the US. Specifically, two experiments were conducted to analyze the effect of manipulating total time of context-drug pairings (Experiment 1) and the duration of the Inter Stimulus Interval between drug administration and context exposure (Experiment 2). We recorded the time that the animals remained in an unusual position using the bar test as an index of catalepsy (see for example, Sanberg et al., 1996). If catalepsy conditioning corresponds to the rules of Pavlovian conditioning, there should be an increase in catalepsy on the drug-free tests when the time of CS–US exposure during conditioning is extended, while conditioned catalepsy should be reduced after an increase in the duration of the interval stimulus interval.

## Experiment 1

The procedure most frequently employed to assess the haloperidol-induced catalepsy response is the bar test (e.g., Sanberg et al., 1996; Alvarez-Cervera et al., 2005). In this procedure, the animal’s front forepaws are placed on a bar elevated above the ground so that the animal is in an unusual position, and the time it takes to lower the forepaws to the ground is recorded. Extended descent latency is considered as an index of catalepsy. In a standard bar test procedure, the animal is administered with the drug (typically a dose of between 0.25 and 1 mg/kg of haloperidol), and then, within 10–60 min, the bar test is applied, and the animal is returned to its home cage. In most of the published experiments, this treatment is repeated for 8–10 days, at a rate of one trial per day, after which a free-drug test trial is given to evaluate the magnitude of conditioned catalepsy (e.g., Amtage and Schmidt, 2003; Banasikowski and Beninger, 2012).

A peculiarity of the experiments in which drug effects are used as the US compared to other Pavlovian procedures is the temporal relationship established between the CS and the US. While in most Pavlovian procedures, the CS is presented before the appearance of the US, when the US is the effect of a drug the US is administered before CS presentation, but the effects of the US are experienced simultaneously to CS exposure. Therefore, such arrangement between the CS and the US is a combination of backward and simultaneous conditioning. In the case of delayed or trace conditioning, in which the CS precedes the onset of the US, the number of pairings becomes a critical factor in determining the intensity of the association and thus the conditioned response (e.g., Rescorla and Wagner, 1972; Mackintosh, 1975). However, in the case of simultaneous conditioning, in addition to the number of pairings, a second factor determining the strength of the association between the two stimuli is the length of time for which the two stimuli are paired, such that, as this duration increases the intensity of the association increases.

Therefore, the purpose of this experiment was twofold. First, we aimed to induce conditioned catalepsy by repeatedly pairing a distinctive new context (CS) with the effect of haloperidol administration (US). Second, we compared the strength of conditioning produced by short vs. long exposure to the context-CS following US administration. To this end, we used a 2 × 2 factorial design with the main factors of Conditioning (Paired vs. Unpaired) and Context exposure (1 min vs. 1 h). With this arrangement, half of the animals received four conditioning trials consisting of an injection of haloperidol before context exposure (Paired – Pair condition), while the other half received the drug after context exposure (Unpaired – Unp condition). In addition, half of the animals in each conditioning condition were removed from the CS-Context immediately after the catalepsy test (“short” condition), and the other half remained in the CS-Context for one additional hour after the bar test ended (“long” condition).

Our hypotheses anticipate that those animals in the Paired condition will develop an association between the context and the effects of haloperidol that will be expressed as conditioned catalepsy during the free-drug test. Additionally, for those animals in the long exposure condition, we anticipate more intense conditioned catalepsy at testing than the animals in the short exposure condition.

### Materials and Methods

#### Subjects

Thirty-two experimentally naïve male Wistar rats (*n* = 8), participated in this experiment. At the arrival to the laboratory, the animals were housed in groups of 2/3 (depending of the animals’ weight) in type IIIH cages (820 cm^2^), with wood savings as bedding and other materials available in the cages (pieces of fabric, cardboard and wood, stones, etc.), except for the time they were submitted to the experimental procedure when they were individually housed. The mean weight at the start of the experiment was 357 g (range 300–450 g). Food and water were available *ad libitum* throughout the experiment. During the experimental period each animal was individually housed and maintained on a 12:12 h light–dark cycle (lights on at 07:00 h). All behavioral testing was conducted during the light period of the cycle. Four days before the start of the experimental sessions, each of the animals was handled daily for 5 min. All procedures were conducted in accordance with the guidelines established by the EU Directive 2010/63/EU for animal experiments, and the Spanish R.D. 223/1988.

#### Apparatus and Drugs

For those animals in the “long” exposure condition, four identical Panlab conditioning boxes (model LE111, Panlab/Harvard Apparatus, Spain) were used, each measuring 26 × 25 × 25 cm (H × L × W) and enclosed in a sound-attenuating cubicle (model LE116. Panlab/Harvard Apparatus, Spain). A horizontal bar was attached to the walls of the experimental chamber (diameter, 1 cm; length, 25 cm; 10 cm above the test platform). Catalepsy was recorded by measuring descent latency (the time that elapsed from the animal placing its forepaws on the bar until both forepaws touched the floor) using a stopwatch. Those animals in the “short” exposure condition were submitted to conditioning and testing in four automated catalepsy test chambers (Med Associates, Inc., St. Albans, VT, United States). The rat’s forepaws were placed on a horizontal cylindrical metal bar (diameter, 1 cm; length, 15 cm; 10 cm above the test platform), and the instrument measured the time that contact was maintained between the floor and the bar. A cut-off time of 60 s was used for all animals in every condition. The drug injected was Haloperidol (Kern Pharma), administered subcutaneously in the nape of the neck at a dose of 0.5 mg/kg. A saline solution was used as vehicle. All experimental procedures were initiated 20 min after the drug was injected, since this is the time at which maximum concentration of the drug in the plasma following dose administration is reached (Kudo and Ishizaki, 1999).

#### Procedure

The animals were divided into four groups: Pair/Short, Unp/Short, Pair/Long, and Unp/Long. Those animals in the Pair condition received the corresponding drug 20 min before being introduced into the experimental context, and the vehicle 20 min after being returned to their home cages; those rats in the Unp condition received the vehicle 20 min before experimental context exposure, and the drug 20 min after they were removed from the experimental chamber.

The experiment started with a baseline trial with the animals being injected with saline solution 20 min before to register descent latency in the bar test. Each animal was immediately returned to its home cage after this trial. The next day started the context conditioning phase that consisted of one daily session during four consecutive days. On each session descent latency for each animal was registered. Those animals in the Short exposure condition were immediately returned to their home cages after the catalepsy bar test finished. The animals in the Long exposure condition remained in the test context for one additional hour. After 2 days without treatment, intended to eliminate any possible residual drug in the animal’s system, a single drug-free test was conducted (also initiated 20 min after saline administration). Descent latency was registered as an index of catalepsy.

### Results

#### Baseline

Mean descent latency at baseline was 1.16 s (Range 0.7–3.52). A 2 × 2 ANOVA with main factors Conditioning (Pair vs. Unp) and Context exposure (Short vs. Long) conducted on mean descent latency at the baseline day revealed that neither the main effects nor the interaction was significant (all *p*s > 0.13).

#### Context Conditioning

**FIGURE 1:**
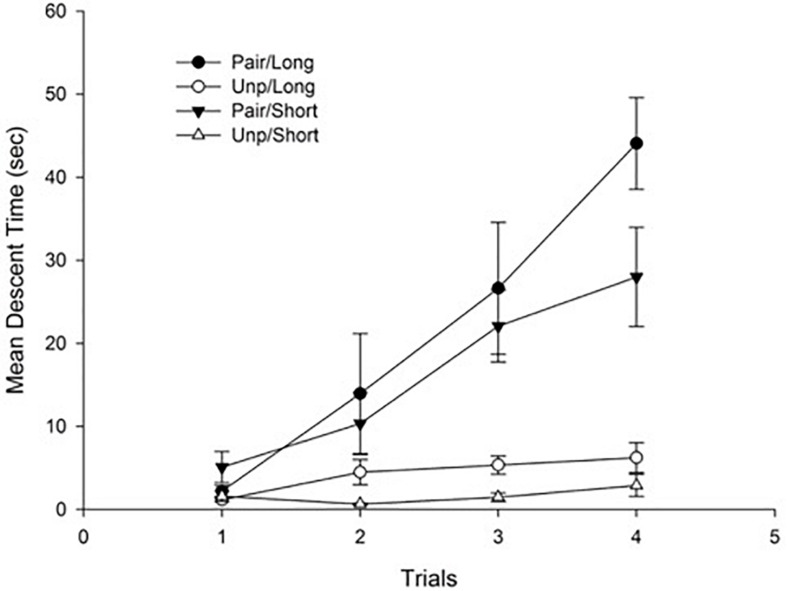
Mean descent latency for conditioning trials as a function of Groups. Pair: Paired; Unp: Unpaired. Error bars represent standard error of the mean (SEM)s.

Figure 1 depicts mean descent latency as a function of groups. As can be seen in the figure, haloperidol-induced catalepsy appeared for those animals in the Pair condition, and it increased across trials reflecting a sensitization process.

These impressions were confirmed by the statistical analyses. Specifically, a 4 × 2 × 2 mixed ANOVA with main factors Trials (within-subject), Conditioning (Pair vs. Unp), and Context exposure (Short vs. Long) conducted on mean descent latency revealed a significant main effect of Trials, *F*(3, 84) = 18.67; *p* < 0.001, η^2^ = 0.40, reflecting an overall increase in descent latency across trials. The main effect of Conditioning was also significant, *F*(1, 28) = 56.44; *p* < 0.001, η^2^ = 0.66, due to the higher mean descent time for the Pair as compared to the Unp Group (Mean = 19.04 s, SD = 8.58, and Mean = 2.97 s, SD = 2.04, respectively). Finally, the Trials × Conditioning interaction was also significant, *F*(3, 84) = 12.79; *p* < 0.001, η^2^ = 0.31. The interaction reflects the progressive increase in descent latency that was evident only for those animals in the Pair condition. No more main effects or interactions were significant (all *p*s > 0.05).

#### Free-Drug Test

**FIGURE 2:**
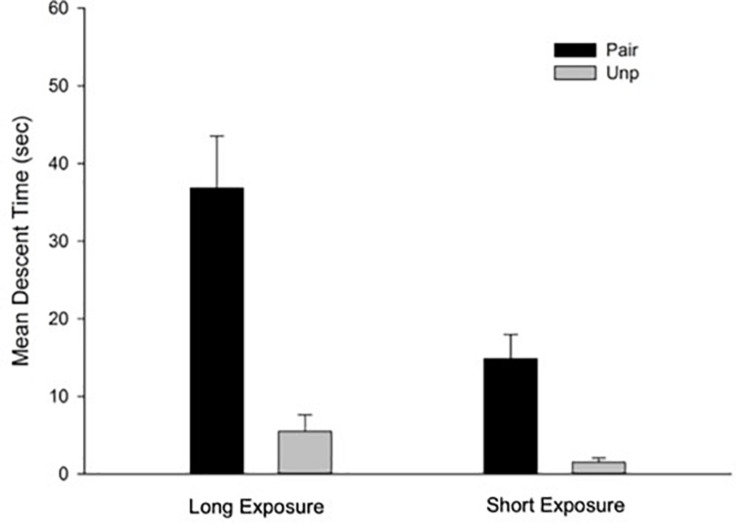
Mean descent latency for the drug-free test trial as a function of Groups for the free-drug test trial. Pair: Paired; Unp: Unpaired. Error bars represent SEMs.

Mean descent time for the free-drug test trial as a function of Groups is depicted in Figure 2. A 2 × 2 ANOVA with main factors Conditioning and Context exposure revealed significant main effects of Conditioning and Context exposure, *F*(1, 28) = 33.65; *p* < 0.001, η^2^ = 0.55, and *F*(1, 28) = 11.37; *p* < 0.01, η^2^ = 0.29, respectively. The main effect of Conditioning was due to a general conditioned catalepsy effect in the Pair as compared to the Unp groups (Mean = 25.85 s, SD = 18.23, and Mean = 3.51 s, SD = 4.7, respectively). The main effect of Context exposure was due to higher mean descent latency for the animals in the Long exposure as compared to those in the Short exposure condition (Mean = 21.17 s, SD = 21.1, and Mean = 8.18 s, SD = 9.25, respectively). Interestingly, the two-way interaction was also significant, *F*(1, 28) = 5.46; *p* < 0.05, η^2^ = 0.16. Between groups *post hoc* comparisons (Bonferroni, *p* < 0.05) performed to identify the source of the interaction revealed significant differences between the Pair/Long and the Unp/Long groups, indicating the predicted effect of conditioned catalepsy. However, the difference between the Pair/Short and the Unp/Short was non-significant. Finally, the difference between Pair/Long and Pair/Short groups was also significant.

## Experiment 2

In Experiment 1, we found that the length of the CS and US pairing is an essential factor in determining the intensity of catalepsy conditioning, since with four pairings of the context-CS and the effect of haloperidol conditioning only appeared when the CS–US exposure time was 1 h (long exposure condition), but not when this exposure was around 1 min (short exposure condition).

In our second experiment, we manipulated an additional temporal variable that has been shown to play an important role in determining the intensity of a Pavlovian association, namely the inter-stimulus interval (ISI), defined as the temporal gap between presentation of the CS and the US. Previous research on this issue using traditional Pavlovian procedures has demonstrated that the longer the interval, the weaker the association (e.g., Smith et al., 1969; Yeo, 1974; Bevins and Ayres, 1995; Cunningham et al., 1997). However, in the case where drugs are used as the US, the ISI presents peculiarities that make this type of paradigm different from traditional Pavlovian procedures, since in the first case, the US (the drug) is administered first, and then the CS-context is exposed, whereas in the traditional procedure the CS is presented before the US. However, we can anticipate that conditioning will be stronger if the CS is present when the drug reaches its maximal level in the brain (Broadbent and Cunningham, 1996). Since previous research has revealed that haloperidol concentrations reach their maximum level in the rodent brain 15 min after administration after which it slowly declines beyond this time during the first 6 h (e.g., Kapetanovic et al., 1982; Zetler and Baumann, 1985), we can estimate that the intensity of the US during exposure to the context on conditioning trials will be higher for a shorter ISI.

Therefore, in the present experiment, we evaluated whether the intensity of conditioned catalepsy after repeated administration of 0.5 mg/Kg of haloperidol in the presence of specific context-CS decreases when the ISI is increased. To this end, we employed a design similar to that described for Experiment 1 for the Pair and Unp groups in the Long exposure condition with an ISI of 20 min (Pair/20 and Unp/20), but we introduced two further groups with a 90-min ISI (Pair/90 and Unp/90). We expected more intense conditioned catalepsy for the Pair/20 Group than the Unp/20 group and a reduction in conditioning for the Pair/90 group.

### Materials and Methods

#### Subjects

Thirty-two experimentally naïve male Wistar rats (*n* = 8), participated in this experiment. The mean weight at the start of the experiment was 348 g. (Range 304 – 417 g). The animals were housed and maintained as described for Experiment 1. All procedures were conducted in accordance with the guidelines established by the EU Directive 2010/63/EU for animal experiments, and the Spanish R.D. 223/1988.

#### Apparatus and Drugs

The apparatus and drugs were the same as described for those animals in the long exposure condition from Experiment 1.

#### Procedure

The animals were divided into four groups: Pair/20, Unp/20, Pair/90, and Unp/90. Those animals in the Pair/20 and Unp/20 groups were treated exactly as described for the Pair/Long and Unp/Long groups from Experiment 1. The same treatment was applied to the Pair/90 and Unp/90 groups, except for the time between the solution injected (Hal or Sal) and the start of the bar test, that was 90 min. In addition, in this experiment the free-drug test trial was conducted for three consecutive days in order to check a possible extinction effect.

### Results

#### Baseline

Mean descent latency at baseline was 1.96 s (Range 0.32–13.70). A 2 × 2 ANOVA with main factors Conditioning (Pair vs. Unp) and ISI (20 vs. 90) conducted on mean descent latency the baseline day revealed that neither the main effects nor the interaction was significant (all *p*s > 0.38).

#### Context Conditioning

**FIGURE 3:**
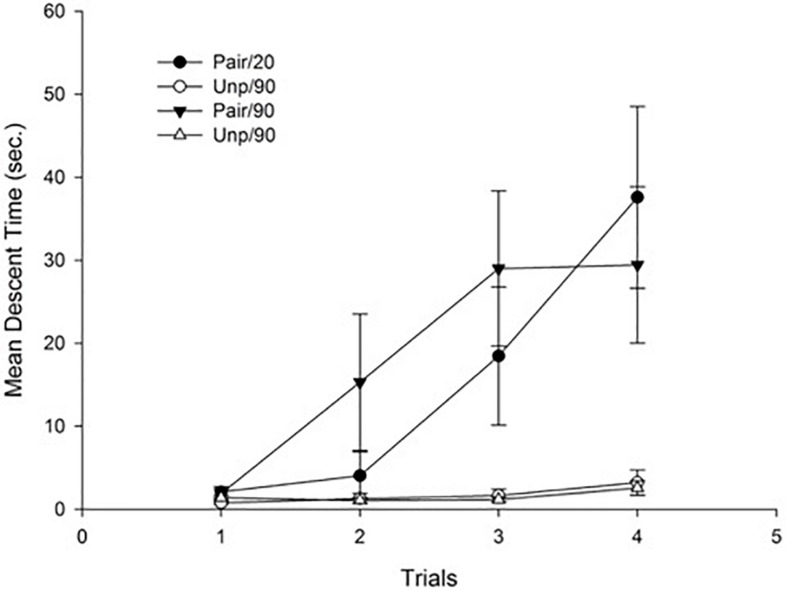
Mean descent latency for conditioning trials as a function of Groups. Pair: Paired; Unp: Unpaired. Error bars represent SEMs.

Figure 3 depicts Mean descent latency as a function of groups. As can be seen in the figure, there was a progressive increase of catalepsy in the Paired groups, reflecting a sensitization effect of repeated haloperidol administration that was irrespective of the ISI.

This impression was confirmed by the statistical analyses. Specifically, a 4 × 2 × 2 mixed ANOVA with main factors Trials (within-subject), Conditioning (Pair vs. Unp), and ISI (20 min vs. 90 min) conducted on mean descent latency revealed significant main effects of Trials and Conditioning, *F*(3, 84) = 9.12; *p* < 0.001, η^2^ = 0.25, and *F*(1, 28) = 24.86; *p* < 0.001, η^2^ = 0.47, respectively. The main effect of Trials reflects an overall increase of mean descent time across trials. The effect of conditioning was due to a higher mean descent time for the Pair as compared to the Unp groups (Mean = 17.22 s, SD = 12.12, and Mean = 1.63 sc, SD = 1.48, respectively). The Trials × Conditioning interaction was also significant, *F*(3, 84) = 7.42; *p* < 0.001, η^2^ = 0.21, reflecting an increase across trials of mean descent latency that was restricted to the Pair groups. No more main effects or interactions were significant (all *p*s > 0.44).

#### Free-Drug Test

**FIGURE 4:**
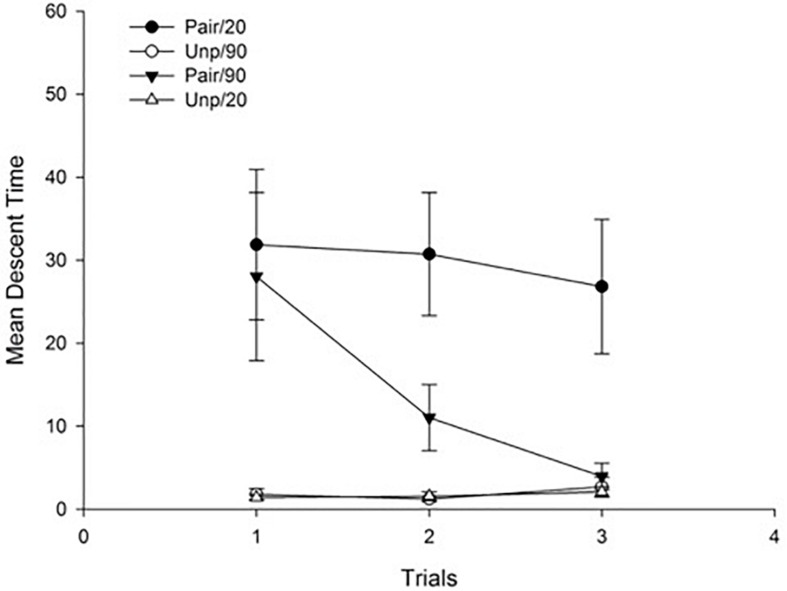
Mean descent latency for drug-free test trials as a function of Groups. Pair: Paired; Unp: Unpaired. Error bars represent SEMs.

Figure 4 depicts mean descent time for each free-drug test trial as a function of Groups. As can be seen in the figure, the effect of conditioned catalepsy appeared in both paired groups for the first test trial, irrespective of the ISI, but it extinguished faster across trials in the Pair/90 as compared to the Pair/20 Group.

A 3 × 2 × 2 ANOVA with main factors Trials (within-subject), Conditioning (Pair vs. Unp), and ISI (20 min vs. 90 min) was conducted on mean descent latency for the free-drug test trials. The main effects of Trials and Conditioning were significant *F*(2, 56) = 6.39; *p* < 0.01, η^2^ = 0.19, and *F*(1, 28) = 18.64; *p* < 0.001, η^2^ = 0.40, respectively, reflecting an overall reduction of mean descent time across trials, and higher descent times for the animals in the Pair as compared to those in the Unp condition (Mean = 22.07 s, SD = 19.80, and Mean = 1.80 s, SD = 1.03, respectively). Neither the main effect of ISI nor the Conditioning × ISI interaction was significant (both *p*s > 10). All interactions involving trials were significant: The Trials × Conditioning interaction, *F*(2, 56) = 7.66; *p* < 0.01, η^2^ = 0.22, reflects an overall decrease in descent latency across trials for those animals in the Pair groups. The Trials × ISI interaction, *F*(2, 56) = 3.41; *p* < 0.05, η^2^ = 0.11, was due to a sharper reduction in descent latency across trials for the animals in the 90 min as compared to those in the 20 min condition. Finally, in order to identify the source of the Trials × Conditioning × ISI interaction, *F*(2, 56) = 3.32; *p* < 0.05, η^2^ = 0.11, we conducted simple effect analyses (*t*-tests, *p* < 0.05) on each trial that revealed a significant effect of conditioned catalepsy when comparing Pair/20 vs. Unp/20 groups for all three trials, while the same effect was significant only for the first trial when comparing the Pair/90 vs. Unp/90 Groups. Therefore, and as can be seen in Figure 4, conditioned catalepsy was extinguished from the second test when the ISI was 90 min, but it remained intact across trials when the ISI was of 20 min.

## General Discussion

The results of Experiments 1 and 2 were consistent with those of previous reports showing that pairing the effects of haloperidol (US) with a neutral context (CS) results in conditioned catalepsy (e.g., de Sousa Moreira et al., 1982; Banasikowski and Beninger, 2012). Additionally, an extended period of exposure to the Context-CS and the effect of the drug (Exp. 1), and a reduced ISI between the CS and the US (Exp. 2), two experimental manipulations that have been shown to intensify the strength of the CS–US association in other conditioning preparations, were effective in increasing conditioned catalepsy.

Although the temporal relationships between CS and US have been considered an essential factor in the establishment of conditioning and the intensity of the CR (e.g., Savastano and Miller, 1998; Kirkpatrick and Balsam, 2016), in situations in which drugs are used as the US, the effects of these types of variables have not been analyzed in detail. This could possibly be related to the unconventional temporal relationships established in drug experiments since, in these cases, the CS and the effect of the drug are experienced simultaneously. In contrast, in traditional procedures, the CS is presented before the US, and conditioning is considered to occur when the CS acquires predictive value concerning the appearance of the US (e.g., Rescorla, 1972). In fact, traditional experiments that have employed simultaneous conditioning procedures have yielded contradictory results since in some studies no conditioning has been observed (e.g., Smith et al., 1969), while others have found evidence of associations between the CS and US (e.g., Barnet et al., 1991).

To our knowledge, there are no systematic studies that have analyzed the effect of trial duration on the intensity of conditioned catalepsy, although there is evidence from experiments in which drugs have been used as US to induce place preference conditioning (see for a review, Tzschentke, 2007). In this type of experiment, animals are exposed to two different experimental contexts, one of which is experienced simultaneously with the effects of a drug with rewarding effects (the US), while in the other, the US is never presented. Following conditioning, a drug-free test is given in which animals are allowed access to the two contexts. An increase in the amount of time spent in the conditioned context is taken to indicate the rewarding properties of the CS (e.g., Bardo and Bevins, 2000). In a meta-analysis conducted by Bardo et al. (1995), it was concluded that in place preference experiments using morphine, amphetamine, heroin, or cocaine, conditioning is stronger as the duration of the conditioning trials increases. In line with these results, in our first experiment, we have found that for a given number of conditioning trials, the duration of exposure time to the context in the presence of the drug is a fundamental factor that determines the strength of conditioning. Thus, we can conclude that in this type of experiment, the intensity of the CS–US association will be determined by the number of pairings (e.g., Rescorla and Wagner, 1972), but there will also be a positive correlation between the time of exposure to the CS–US episode and the intensity of conditioning.

Concerning the manipulation of the time interval between the CS and the US, this variable has a marked effect on the intensity of conditioning in traditional experimental procedures, such that as the interval increases, the association between the CS and the US weakens (e.g., Yeo, 1974). As we mentioned in the introduction to Experiment 2, in the case of conditioning with a drug US, the temporal relationship between the CS and the US is different from that produced in traditional procedures. However, we can anticipate experiencing the CS when the concentration of the drug in the brain is maximal will favor the establishment of a stronger CS–US association (Broadbent and Cunningham, 1996). To our knowledge, no studies have systematically analyzed the effect of the ISI on conditioning using haloperidol. However, inspection of the ISIs used in previously published experiments pairing haloperidol administration with CS-context exposure shows a range of time intervals varying between 10 min (e.g., Oliveira et al., 2016), 15 min (e.g., Colombo et al., 2013), or 60 min (e.g., Banasikowski and Beninger, 2012), all of which were successful in producing conditioned catalepsy.

The results of Experiment 2 revealed that the increase from 20 to 90 min of the interval between haloperidol administration and the onset of context exposure did not affect the catalepsy CR on the first test trial. In contrast, a reduction of conditioned catalepsy was observed in the group with the longer ISI, since extinction of the CR was faster for the Pair/90 group than the Pair/20 group. The fact that no differences appeared between the two matched groups on the first test trial is logical if we consider the strong catalepsy effect induced by haloperidol during conditioning regardless of the ISI used (Figure 3); in fact, although the concentration of haloperidol in the brain starts to decline 15 min after administration, the effect on the motor response persists for up to 6 h (e.g., Zetler and Baumann, 1985).

There are two possible reasons why conditioning was extinguished more rapidly in the group with the longer ISI. The first is the lower brain drug concentration present during exposure to the context on the conditioning trials for the Pair/90 group compared with the Pair/20 group (Broadbent and Cunningham, 1996). The second reason could be related to the possibility that when the animals remain in their home cages between haloperidol administration and introduction into the experimental context, this could interfere with conditioning. Specifically, while animals in the Pair/20 group remained for 20 min in their respective home cage and 60 min in the context-CS for each of the conditioning trials, animals in the Pair/90 group remained for 90 min in the home cage following haloperidol administration and 60 min in the context-CS. This difference could have resulted in an interference process whereby the association between the home cage and the effect of the drug would have been more firmly established in the Pair/90 group than the Pair/20 group and, therefore, the associative strength available during exposure to the experimental context would have been lower for the former group than for the latter (e.g., Rescorla and Wagner, 1972). However, regardless of whether the reduction in conditioning observed after the increase in ISI in Experiment 2 is due to a lower intensity of the US experienced during the conditioning trials or an interference effect on conditioning, the results are consistent with a Pavlovian interpretation of the conditioned catalepsy response.

In short, our results are particularly relevant for understanding the process underlying the conditioned catalepsy response, since at least three different explanations have appeared in the literature to explain the results of conditioning using drugs that affect the dopaminergic system. First, some authors have proposed that the responses observed in these types of experiments could be the result of a non-associative process, since the administration of dopamine agonist or antagonist drugs before exposure to the context-CS could hinder context processing, making the context functionally novel at the time of testing and eliciting numerous orienting responses that would have a confounding effect on the observed CR (e.g., Damianopoulos and Carey, 1992). However, this possibility has been ruled out by the results of various experiments employing both dopaminergic agonists and antagonists (e.g., Ahmed et al., 1996; De la Casa et al., 2018, respectively).

A second possibility is related to the rewarding properties of dopaminergic agonist drugs that, after pairing with the context, induce approach responses that would be manifest as increased locomotor activity on the conditioning test (e.g., Beninger and Olmstead, 2000). This perspective considers the rewarding value of drugs such as amphetamine or apomorphine that have usually been employed in this type of experiment, resulting in increased dopaminergic activity in the mesotelencephalic reward system (Banasikowski and Beninger, 2010). This hypothesis, however, could not explain the results in which the drug administered is a dopaminergic antagonist since this type of substance has no rewarding action and has even been shown to block the rewarding value of other stimuli (Spyraki et al., 1982).

Finally, a third interpretation on the origin of the responses observed after pairing the context with dopaminergic modulatory drugs can be established in strictly Pavlovian terms, considering that the CS is a stimulus that acquires the same properties as the US and, therefore, comes to induce an adaptive response that is appropriate to the occurrence of the US (e.g., Mena et al., 2016; Santos et al., 2017). This perspective adopts a dual approach, depending on whether we focus on analyzing observable motor responses or on the underlying physiological processes. Regarding the observable responses, we could consider that the movement-reducing effect of haloperidol would be associated with the context in which catalepsy is measured and would translate into motor-type conditioning that would result in reduced movement on the drug-free test. From a physiological perspective, the primary pharmacological action of haloperidol is the blockade of D2 receptors, some of which are autoreceptors located on terminals and dendrites, while others are located post-synaptically on the soma, dendrites, and terminals of noradrenergic neurons (Osborne et al., 1994). Haloperidol, by blocking presynaptic D2 receptors (autoreceptors), increases dopamine release by preventing the *feedback* exerted by autoreceptors under normal conditions, but the increase in dopaminergic transmission is rendered ineffective by the blockade of postsynaptic D2 receptors (Lidsky I, and Banerjee, 1993). Therefore, it is possible that the physiological component of the CR when haloperidol is used as the US reproduced this postsynaptic D2 receptor blockade that gives rise to conditioned catalepsy. Data supporting this interpretation comes from experiments using locomotor response conditioning with amphetamine as the US, which have reported changes in dopaminergic transmission levels with *in vivo* microdialysis procedures. For example, Dietze and Kuschinsky (1994) found an increase in the extracellular concentration of dopamine in the striatum parallel to recording the locomotor response in the absence of a drug (see also, Schiff, 1982; Fontana et al., 1993). In future studies it would be of interest to analyze changes in dopaminergic activity following haloperidol conditioning, which would help to shed light on the physiological-level conditioning processes that parallel the behavioral changes shown in our experiments.

## Data Availability Statement

The raw data supporting the conclusions of this article will be made available by the authors, without undue reservation.

## Ethics Statement

The animal study was reviewed and approved by Comité Ético de Experimentación Animal (CEEA), Universidad de Sevilla.

## Author Contributions

Both authors listed have made a substantial, direct and intellectual contribution to the work, and approved it for publication.

## Conflict of Interest

The authors declare that the research was conducted in the absence of any commercial or financial relationships that could be construed as a potential conflict of interest.
